# Editorial Comment: Assessment of Factors Responsible for Stone-Free Status After Retrograde Intrarenal Surgery

**DOI:** 10.1590/S1677-5538.IBJU.2025.9901

**Published:** 2025-01-05

**Authors:** Raj K Kishan, Adiga K Prashant, Chandni Clara D’souza Reshmina, B Nandakishore, Manjunath Shetty

**Affiliations:** 1 Father Muller Medical College and Hospital Urology Mangalore IND Urology, Father Muller Medical College and Hospital, Mangalore, IND; 2 Father Muller Medical College and Hospital General Surgery Mangalore IND General Surgery, Father Muller Medical College and Hospital, Mangalore, IND; 3 Malabar Medical College Hospital and Research Centre Urology Kozhikode IND Urology, Malabar Medical College Hospital and Research Centre, Kozhikode, IND

**Cureus. 2024 Jul 1;16(7):e63627**

DOI: 10.7759/cureus.63627|ACCESS: 3895751

## COMMENT

Retrograde intrarenal surgery is a great option to treat renal stones and had low risk of complications compared to percutaneous nephrolithotomy (PCNL). The anatomic aspects are of great importance for these procedures (1). The previous paper shows the importance of pre-operative oral antibiotics to reduce the risk of infection in this surgery (2). In the paper of Raj and collegues (3) the authors evaluate the predictive factors that determined stone-free rate (SFR) after retrograding intrarenal surgery (RIRS). In this prospective study 183 patients undergoing RIRS for renal stones were studied. The authors concluded that RIRS has lately emerged as an effective and reliable modality for treating selected renal stones. Patients with large or multiple stones require follow-up due to the high risk of residual stones after a single session of RIRS. Patients who undergo RIRS in large-volume stones should be counseled for staged procedures beforehand. Lower pole stone location, stone density (HU), and abnormal renal anatomy are essential predictors for SFR after RIRS. Lower pole RIPA and RIL are significant influencing factors for SFR after RIRS. RIRS and RUSS scores show a significant association with stone-free outcomes, with higher scores predicting poorer SFR. RIRS score performed better than the RUSS score in predicting stone-free outcomes. These scoring systems can be used preoperatively to gauge treatment success and counsel patients regarding appropriate treatment modalities. We congratulate the authors for the interesting paper.
